# The Reinforcing Therapist Performance (RTP) experiment: Study protocol for a cluster randomized trial

**DOI:** 10.1186/1748-5908-5-5

**Published:** 2010-01-26

**Authors:** Bryan R Garner, Susan H Godley, Michael L Dennis, Mark D Godley, Donald S Shepard

**Affiliations:** 1 Lighthouse Institute, Chestnut Health Systems, Normal, IL, USA; 2 Schneider Institute for Health Policy, Heller School, Brandeis University, Waltham MA, USA

## Abstract

**Background:**

Rewarding provider performance has been recommended by the Institute of Medicine as an approach to improve the quality of treatment, yet little empirical research currently exists that has examined the effectiveness and cost-effectiveness of such approaches. The aim of this study is to test the effectiveness and cost-effectiveness of providing monetary incentives directly to therapists as a method to improve substance abuse treatment service delivery and subsequent client treatment outcomes.

**Design:**

Using a cluster randomized design, substance abuse treatment therapists from across 29 sites were assigned by site to either an implementation as usual (IAU) or pay-for-performance (P4P) condition.

**Participants:**

Substance abuse treatment therapists participating in a large dissemination and implementation initiative funded by the Center for Substance Abuse Treatment.

**Intervention:**

Therapists in both conditions received comprehensive training and ongoing monitoring, coaching, and feedback. However, those in the P4P condition also were given the opportunity to earn monetary incentives for achieving two sets of measurable behaviors related to quality implementation of the treatment.

**Outcomes:**

Effectiveness outcomes will focus on the impact of the monetary incentives to increase the proportion of adolescents who receive a targeted threshold level of treatment, months that therapists demonstrate monthly competency, and adolescents who are in recovery following treatment. Similarly, cost-effectiveness outcomes will focus on cost per adolescent receiving targeted threshold level of treatment, cost per month of demonstrated competence, and cost per adolescent in recovery.

**Trial Registration:**

Trial Registration Number: NCT01016704

## Background

Alcohol and other drug abuse problems are increasingly being recognized as a chronic, relapsing condition that may last for decades and require multiple episodes of care over many years [1-3]. As over 80% of all people who develop alcohol and other substance use disorders start using under the age of 18 [4], there is clearly a need for effective treatment interventions designed specifically for adolescents. Unfortunately, while a number of effective evidence-based treatments (EBTs) have been developed for treating adolescent substance abuse and dependence [5-14], the diffusion of such EBTs into practice settings has been found to be a significant challenge [15-18].

Since the identification of this important issue, there has been great interest in bridging the 'research-to-practice gap', including research to understand the correlates of EBT adoption [19,20] and staff attitudes toward EBT use [21-23]. Additionally, several conceptual models of the EBT adoption and implementation process have been developed [24-27]. Despite these advances, there remains much room for further improvement, especially in the identification of methods that facilitate implementation of EBTs [18,28,29]. This is a critically important area of research, given meta-analyses of treatment programs have suggested that the degree of implementation can be as important as the nominal efficacy of the targeted EBT, with the biggest effects coming from well-implemented, highly efficacious interventions [30]. In order to reliably achieve effective treatment outcomes, it is necessary to empirically test ways to improve the EBT implementation process in practice settings.

While multiple factors influence the quality and degree of EBT implementation in practice settings, attention has increasingly focused on the role of the therapist as a key mediator of treatment delivery over the last decade [31-34]. Indeed, Walters, Matson, Baer, and Ziedonis [35] conducted a systematic review of the effectiveness of workshop training for psychosocial addiction treatments and concluded that workshop trainings generally improved therapist knowledge, attitude, and confidence in working with clients, as well as some skills immediately after training. However, they also found that these skills typically were not maintained for very long. In order for therapists to incorporate these skills in their repertoire for the long-term, they concluded that extended contact, including feedback, supervision, and consultation, is also necessary. Support for this conclusion is perhaps best provided by the studies that used experimental designs to test different training strategies [33,34]. For example, Miller and colleagues [33] evaluated four methods to help therapists learn motivational interviewing (MI), including: workshop only; workshop plus practice feedback; workshop plus individual coaching; and workshop, feedback, and coaching. Only therapists in the full training condition (*i.e.*, workshop, feedback, and coaching) had clients with significant changes in their response to treatment. However, even these state-of-the-art training and technical assistance strategies may not be enough to ensure quality implementation, as even within carefully controlled clinical trials that employ these strategies, there is often variation in how competently and reliably therapists implement interventions. For example, in an examination of the relationship between therapist competence and clinical outcomes in the Treatment of Depression Collaborative Research Program (TDCRP), Shaw *et al*. [36] found that therapists did not meet the set minimum standard for competence in 27% of sessions. Indeed, in multiple studies that examined this issue, the size of the 'therapist' effect has been as large as or larger than the mean effects between conditions [37-42].

Given therapists are critical in the implementation of high-quality treatment, research is needed to better understand how to improve the degree to which therapists competently deliver EBTs to adolescents. One approach recently recommended by the Institute of Medicine is called pay-for-performance (P4P), and is a variant of contingency management procedures (also called motivational incentives) that have been shown to be effective in the enhancement of a variety of behaviors with alcohol and other substance abusers [43-50]. Interestingly, despite several studies having demonstrated the significant relationship between financial incentives and work performance [51-54], few studies have used randomized clinical trials (RCTs) to examine the impact of P4P initiatives within healthcare [55] or behavioral health [56]. Although not RCTs, there are several notable examples of linking monetary incentives to performance within the substance abuse treatment field [57-59]. For instance, Andrzejewski *et al*. [57] found that providing graphical performance feedback and drawings for cash incentives increased implementation by 69% and 93%, respectively. Shepard *et al*. [58] found that providing therapists with a $100 bonus was an effective and cost-effective approach to improve the percentage of clients who attended five sessions. McLellan *et al*. [59] reported on the Delaware Division of Substance Abuse and Mental Health (DSAMH) 'performance contracting' with all 11 of its outpatient addiction treatment programs. Results indicated that 'capacity utilization' increased from an average of 54% in 2001 to an average of 95% in 2006 and that 'active participation' increased from an average of 53% in 2001 to 70% in 2006. Although P4P methods appear to hold promise for improving treatment implementation, research utilizing rigorous experimental designs and larger sample sizes is clearly needed.

The current paper describes the design and baseline characteristics of the therapists participating in the Reinforcing Therapist Performance (RTP) study, which is a cluster randomized experiment examining the effectiveness and cost effectiveness (CE) of providing monetary incentives directly to therapists as an innovative method to improve treatment service delivery and subsequent treatment outcomes for adolescents and their caregivers. This study is unique in that there are only a handful of studies that focus on staff characteristics and the mechanisms by which staff behaviors are changed, and even fewer randomized experiments in which staff are the unit of analysis.

## Methods

### Overview of conceptual model

**Figure 1:**
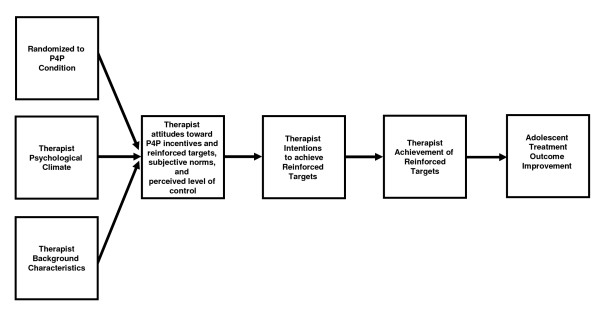
**The conceptual framework for the study**.

Figure 1 illustrates the conceptual framework for the study, which builds upon the Theory of Planned Behavior (TPB) [60] and work by Meterko and colleagues [61]. Specifically, we hypothesize: therapist achievement of the two behaviors being reinforced as part of the study are directly related to their *intentions *to achieve these behaviors and indirectly related (via intentions) to their *attitude toward the incentives, attitude toward the behavior*, *subjective norms *(*i.e.*, social pressure from significant others to engage or not to engage in a behavior, and *perceived level of control *(perceived ease or difficulty of performing a behavior). We also hypothesize that these antecedents of intentions will be directly related to: being randomized to the P4P condition, psychological climate [62] (*i.e.*, therapist perceptions of the organizational climate), and background characteristics (*e.g.*, age, gender, education level, experience). Finally, we hypothesize achievement of the reinforced targets will be associated with improved adolescent treatment outcomes (*e.g.*, reduced substance use).

### Study setting

Consistent with recommendations from a blue ribbon task force on health services research [63], this study represents a unique collaboration between the National Institute on Alcohol Abuse and Alcoholism (NIAAA) and the Center for Substance Abuse Treatment (CSAT). Indeed, the RTP study would not be feasible without the braiding of NIAAA research dollars and more than $30 million dollars from CSAT as part of its Assertive Adolescent and Family Treatment (AAFT) dissemination and implementation initiative. As CSAT's AAFT initiative, which provides the foundational setting for the study, has been described in detail elsewhere (Godley, Garner, Smith, Meyers, & Godley, 2010), only a brief description is provided here.

Between 2006 and 2007, CSAT awarded three-year grants to 34 community-based organizations across the United States to implement a standardized assessment called the Global Appraisal of Individual Needs (GAIN) and two EBTs called the Adolescent Community Reinforcement Approach (A-CRA) and the Assertive Continuing Care (ACC). The latter are EBT adaptations for adolescents of the Community Reinforcement Approach [64] (CRA), and have been shown to be effective in the treatment of adolescent substance abuse and dependence [8,9,65-68]. The purpose of these demonstration grants are to help address the research-to-practice gap by helping community-based treatment agencies implement effective assessment and treatment practices for adolescents and their families/primary caregivers. Based on the research literature and the center's experience that both training and ongoing consultation/coaching are necessary components of successfully implementing EBTs [24,33,34], CSAT also awarded a contract to Chestnut Health Systems to deliver the GAIN and A-CRA/ACC training and technical assistance model to all 34 grantees.

### Overview of RTP study design

This RTP experiment and the rest of this article focus on improving the implementation of A-CRA/ACC. As part of the comprehensive A-CRA/ACC training model received by both RTP groups, participants: read the A-CRA manual and pass a knowledge test prior to training; attending a 3.5-day training workshop; participate in bi-weekly telephone coaching calls with treatment model experts; receive quantitative and qualitative feedback on actual session performance throughout the certification process; receive feedback on actual session performance as part of randomly selected post-certification fidelity checks; and provide documentation of treatment implementation via therapist reports of procedures delivered during each treatment session as well as corresponding digital session recordings (DSRs) of the session. Thus, with 34 grantees across 15 states, the AAFT project represents one of the field's largest dissemination and implementation initiatives of an adolescent substance abuse treatment intervention to date. More importantly, the standardized level of funding and training being delivered to the 34 CSAT grantees provides an ideal setting in which to examine methods to improve implementation.

RTP is a cluster randomized experiment examining the effectiveness and CE of providing monetary incentives to therapists as a method to improve treatment implementation and subsequent outcomes for adolescents and their caregivers. It builds upon prior work by Garner and colleagues [65] that has shown exposure to A-CRA procedures significantly mediates the relationship between treatment retention and outcomes, and empirically identified a threshold level of A-CRA exposure significantly related to positive post-treatment outcomes (*i.e.*, being in recovery). Additionally, it builds upon research that has examined the relationship between therapist competency and treatment outcome for clients [36,69,70]. ACRA/ACC sites and therapists within site were recruited to participate in the study, and those who agreed were randomly assigned to either implementation as usual (IAU) or P4P. Participation was voluntary and the study is conducted under the supervision of Chestnut Health Systems Institutional Review Board (IRB). Below are further descriptions of the intervention, procedures, measures, and analytic plans.

### Study intervention

#### Implementation as usual (IAU)

Both groups receive the same training and technical assistance model they have been receiving since the inception of the AAFT initiative. As noted above, this state-of-the-art training and technical assistance model consists of a 3.5-day workshop training, bi-weekly telephone coaching calls with model experts, and ongoing monitoring and feedback (both quantitative and qualitative) as part of a standardized certification process.

#### Pay-for-performance (P4P)

In addition to the above, the P4P group has the opportunity to earn monetary bonuses for two sets of measurable behaviors related to quality implementation of the model. These two behaviors are: delivering Target A-CRA and demonstrating Monthly A-CRA Descriptions of the rationale and reinforcement schedules for these two targeted behaviors are described in the sections below; however, detailed descriptions of Target A-CRA and Monthly A-CRA competency are provided in the study measures section.

### Rationale and reinforcement schedule for target A-CRA

Research has suggested that the degree of implementation can be as important as the efficacy of the EBT, with the biggest effects coming from well-implemented, highly efficacious interventions [30]. Similarly, our prior research [65] has shown that adolescents who received a threshold exposure of A-CRA were significantly more likely to be in recovery at follow-up. Increasing the number of adolescents who receive Target A-CRA would be expected to result in a higher likelihood that adolescents would have more positive treatment outcomes. Thus, one of the questions the study was designed to examine is the extent to which monetary bonuses could increase the probability that an adolescent receives Target A-CRA. As part of the RTP, study therapists in the P4P condition receive a $200 bonus for each adolescent who receives Target A-CRA within the first 14 weeks of AAFT and in no fewer than seven A-CRA sessions. In order to attribute improvements in adolescent outcomes to the incentives, only outcome data from adolescents admitted to the AAFT project after sites were randomly assigned to the study conditions will be used in Target A-CRA-related analyses.

### Rationale and reinforcement schedule for monthly A-CRA competency

In addition to reinforcing exposure to a threshold number of procedures, we believed it was important to reinforce the quality of delivery (*i.e.*, competence). Thus, P4P therapists also are provided the opportunity to earn a $50 bonus for each month that a randomly selected session recording has at least one core procedure rated at or above the minimum level of competence required for certification. Importantly, in order to ensure a representative sample of session recordings, only those therapists who submit at least 80% or more of treatment session recordings are eligible to have a session rated for competence. Because it would take approximately three months after randomization before P4P participants would be eligible to begin receiving their first bonus associated with delivery of Target A-CRA, reinforcing Monthly A-CRA competency is important as it can be reinforced sooner and more frequently.

### Recruitment

The initial recruitment period for the study occurred between November 2008 and February 2009 and was limited to the sites and therapists participating in CSAT's AAFT initiative. Since the two cohorts of AAFT were funded in different years, recruitment of the study sites was in months 27 and 15 of the cohorts' respective 36-month grants. Although the site's therapists were the target population for the RTP, it was necessary to first obtain permission from each grantee's principal investigator (PI) and/or treatment agency director.

### Site recruitment

Recruitment of study sites began in November 2008. AAFT grantees were first introduced to the study via an email briefly explaining the goals of the study and the extent of involvement the study would require. Email attachments included: the memorandum of understanding, which outlined the responsibilities of the study sites, the informed consent, which outlined the responsibilities of the therapist participants, and a signed letter of support from the CSAT project officer. The study PI (BRG) followed up the e-mail introductions with telephone calls with each site PI to answer questions and inquire about the site's willingness to participate in the study. Out of the 34 grantees, two were excluded for study participation because they were not providing services in an outpatient setting, and two were ineligible because they could not be matched to a comparable site for randomization. Of the 30 eligible grantees, 29 (97%) agreed to participate by returning signed copies of the memorandum of understanding.

### Staff recruitment

Recruitment of therapist participants for the study began one month after site recruitment. In order to be eligible to participate in the study, therapists had to work at one of the participating AAFT grantee sites and be delivering A-CRA or ACC to adolescents. Study packets containing a cover letter, informed consent, staff survey, and a W-9 tax form were mailed to 92 eligible therapists. Of these, 82 (89%) agreed to participate.

### Randomization

Although random assignment of therapists might appear ideal, a number of issues made such an approach impractical and led to the decision to randomize in clusters by site. For example, dividing small (two- to four-person) clinical teams within a site through random assignment may lead to unintended consequences due to some therapists being eligible for incentives and others not. For example, the IAU group might work harder than they normally would to achieve goals (*i.e.*, compensatory rivalry), which would threaten the study's internal validity (increasing type 2 error probability). Another possibility is that this situation would lead to resentful demoralization of therapists in the control group, and they would deliver sub-par effort (increasing type 1 error probability). In order to avoid these potential problems, we used an adaptive randomization procedure referred to as urn randomization [71,72] to assign sites to the two study conditions. Shadish, Cook, and Campbell [71] recommend using such adaptive procedures whenever feasible and when good matching variables can be found, and have noted that the best matching variables are pre-test scores on the outcomes of interest.

Given the two cohorts of AAFT grantees were in months 27 and 15 of their respective 36-month grants, pre-test data was available on several important matching variables. Using existing project data on therapists performance and from staff questionnaires (described further below), we created several grantee-level measures including: average Target A-CRA rate; average DSR upload rate; three-month client recovery rate; percentage of Caucasian clients; percentage of Hispanic clients; percentage of male clients; number of therapists; average therapist age; percentage of Caucasian therapists; percentage of male therapists; and AAFT staff ratings of expected performance. This last measure was used to take into account any recent changes (*e.g.*, turnover of supervisor, major improvement/decrements in performance) that might impact performance in the study, and was based upon independent rankings from the director and coordinator of the AAFT training team. Both raters agreed on the rankings for all but two study sites (Kappa = 0.86), and the two raters were able to discuss and resolve these two inconsistencies. Each of the above-mentioned existing measures was then entered by AAFT cohort into an urn randomization software program called gRand.

Although urn randomization was conducted at the site level, it resulted in a balanced distribution of therapists into the two study conditions (See Table 1). Of the 82 therapists used to randomize sites most were female (74.4%) and Caucasian (56.1%). They had an average age of 37 years (SD = 11.6). In terms of their education and work experience, most had either a Masters (52.4%) or a Bachelor's (41.5%) degree, with an average of 4.3 years of substance abuse counseling experience. Seven percent reported personally being in recovery for alcohol or other drugs. Based on therapist self-report, the average achievement of Target A-CRA implementation prior to the experiment was 19.2%, and the average session recording rate of fidelity was 41.0%. Based on three-month post-intake follow-up data prior to the experiment, the average percentage of therapists' adolescent clients in recovery was 45.9%. Notification to sites and individual participants about the official commencement of the study and their assignment to either the IAU or P4P conditions were sent via email on 16 January 2009 for the AAFT-1 and on 13 February 2009 for AAFT-2.

**Table 1:** Baseline characteristics of therapists at randomization

	P4P (n = 42)	IAU (n = 40)	Overall (N = 82)
	% or M (SD)	% or M (SD)	% or M (SD)
**Age**	36.7 (11.3)	36.7 (12.2)	36.7 (11.6)
			
**Race**			
American Indian/Alaska Native	2.4%	2.5%	2.4%
Asian	0.0%	5.0%	2.4%
African American	11.9%	17.5%	19.5%
Caucasian	52.4%	60.0%	56.1%
Hispanic/Latino	31.0%	15.0%	23.2%
Other	2.4%	0.0%	1.2%
			
**Gender**			
Male	19%	32.5%	25.6%
Female	81%	67.5%	74.4%
			
**Education**			
Less than Bachelor's Degree	0.0%	7.5%	3.7%
Bachelor's Degree	47.6%	35.0%	41.5%
Master's Degree	50.0%	55.0%	52.4%
Doctoral Degree	2.4%	2.5%	2.4%
			
**Years of SAT Experience**	3.3 (3.3)	5.4 (7.1)	4.3 (5.6)
			
**Self-reported being in recovery**	7.1%	7.5%	7.0%
			
**Pre-RTP Target A-CRA Rate**	21.4%	16.7%	19.2%
			
**Pre-RTP Session Recording Rate**	40.4%	41.7%	41.0%
			
**Pre-RTP 3-month Client Recovery Rate**	46.5%	45.1%	45.9%

Note: No statistically significant differences between conditions

### Study measurements

Given that the primary aims of the study were to examine the effectiveness and CE of providing monetary incentives to therapists as a method to improve treatment implementation and subsequent outcomes for adolescents and their caregivers, it was necessary to collect measures from multiple levels (*i.e.*, therapist, adolescent, and grantee) and over several different time points.

### Therapist background and attitude measures

As noted previously, all study participants completed a staff survey at the time of consenting to participate. This 15-page survey took approximate 30 to 45 minutes and asked questions about the individual and the therapist's work environment. Examples include basic socio-demographic characteristics such as age, race, and gender; highest educational degree obtained; and years of substance abuse counseling experience. The survey also included the Minnesota Satisfaction Questionnaire (MSQ) [73], the Pay Satisfaction Questionnaire (PSQ) [74], several scales from the Organizational Readiness for Change (ORC) instrument [75], and several measures adapted from the Provider Attitudes toward Incentive (PAI) [61] instrument. Assessment of changes in participants' attitudes and work environments was measured via three-month follow-up versions of the survey.

### Therapist implementation measures

The two implementation measures being reinforced as part of the study are Target A-CRA and Monthly A-CRA Competency. Developed using existing AAFT data, Target A-CRA is a dichotomous (1 = yes, 0 = no) measure. It is defined as the delivery of 10 or more of the following 12 A-CRA procedures: functional analysis of substance using behavior; functional analysis of prosocial behavior; happiness scale; treatment plan/goals of counseling; communication skills; problem solving skills; adolescent-caregiver relationship skills; caregiver overview, rapport building, and motivation; homework reviewed; drink/drug refusal skills; relapse prevention; and increasing prosocial recreation during the first 14 weeks of an adolescent's AAFT treatment experience (but in no fewer than seven sessions). See the A-CRA treatment manual for a description of these A-CRA procedures [76]. Additionally, because identifying, discussing, and reviewing the adolescent's reinforcers is considered a central mechanism of change within the A-CRA philosophy, as part of Target A-CRA, therapists also must demonstrate one of these three components in at least 50% or more of the sessions conducted during this time period. Therapist-reported data on more than 450 adolescents uploaded to AAFT's implementation tracking system (*i.e.*, https://www.EBTx.org) indicated adolescents who received Target A-CRA had significantly (*p *< 0.05) greater reductions in days abstinent at both three- and six- month post-intake assessments. Importantly, although therapist reports are used to identify adolescents who appear to have received Target A-CRA, official achievement of Target A-CRA for the study requires independent verification (via listening to DSR) by a trained A-CRA rater. See Garner, Barnes, and Godley [77] for complete details regarding the training process for A-CRA raters.

Monthly A-CRA Competency is a dichotomous (1 = yes, 0 = no) measure and indicates whether or not a randomly selected session recording was rated at or above the minimum level of competence required for A-CRA certification (*i.e.*, rating of 3 or higher on all components of the procedure). As described in the A-CRA coding manual [78], each component of an attempted A-CRA procedure is rated using the following categories: 1 = poor, 2 = needs improvement, 3 = satisfactory, 4 = very good, and 5 = excellent. To ensure a representative sample of session recordings, only those therapist participants who submitted at least 80% or more of treatment sessions (minimum of five sessions per month) are eligible to have a session randomly selected and rated for competence. This requirement was implemented in order to reduce the risk of therapists trying to manipulate the criterion being reinforced by only uploading those sessions they expected would pass the competency rating.

### Adolescent intake and follow-up measures

In addition to examining the extent to which monetary incentives improve treatment implementation (*i.e.*, delivery of Target A-CRA, demonstration of Monthly A-CRA Competency), a third aim of the RTP study is to examine the extent to which these two implementation measures impacted treatment outcome for the adolescent clients. Being 'in recovery' (*i.e.*, no past month alcohol or other drug use, abuse, or dependence symptoms while living in the community) was selected as the primary outcome of interest, as is consistent with the primary clinical outcome used in the Cannabis Youth Treatment (CYT) study [8]. Intake and follow-up versions of this measure were collected using the GAIN [79], which is a comprehensive biopsychosocial assessment designed to integrate research and clinical assessment into one structured interview. The GAIN's main scales have been shown to demonstrate good internal consistency (alpha greater than 0.90 on main scales, 0.70 on subscales), test-retest reliability (Rho greater than 0.70 on days/problem counts, kappa greater than 0.60 on categorical measures), and to be highly correlated with measures of use based on timeline follow-back methods, urine tests, collateral reports, treatment records, and blind psychiatric diagnoses (rho of 0.70 or more, kappa of 0.60 or more) [79-81]. GAIN data were collected as part of the AAFT project's evaluation and were de-identified prior to being used as part of the RTP study. In order to access this data, the study group sought and received a signed data sharing agreement from each site that explicitly allowed the use of the de-identified adolescent data for the purposes of research, public health, or healthcare operations.

### Cost measures

Parallel to the RTP study's effectiveness-related aims is a set of aims related to CE. The primary focus of the economic analyses is to compare the operating and reinforcement costs between the IAU and the P4P groups. Operating costs are defined as costs associated with treatment delivery, and reinforcement costs are defined as costs associated with reinforcing superior delivery/implementation of treatment. Additionally, in order to be able to better interpret the findings, it also was necessary to collect information on training costs.

The Treatment Cost Assessment Tool (TCAT) was used to determine operating costs of delivering A-CRA and ACC at each participating AAFT site. The TCAT was developed by Brandeis University in collaboration with Texas Christian University [82] and is an extension of the methods used in the Cost Study of the Alcohol and Drug Services Study (ADSS) [83,84]. The study's TCAT version is a Microsoft^® ^Excel-based workbook that is used to collect information related to a site's clinical activity (*e.g.*, number of clients served, average direct time per treatment session), personnel costs (*e.g.*, percentage of time spent on clinical activities, salary), and non-personnel costs (*e.g.*, supplies, transportation). In contrast to the operating costs, the reinforcement costs are the costs associated with providing the monetary incentives to therapists as part of the RTP study, and are calculated as a total of the payments themselves times (1 + overhead rate of Chestnut Health Systems). The overhead cost is included to reflect the resource costs in administering incentives (*e.g.*, verifying incentive criterion and documenting payments). In the course of completing the TCAT, we will gather data about the persons, steps, and time involved in administering incentives, so we can refine the estimate of administrative costs. Because the clinical training for the AAFT initiative is being funded through a separate CSAT contract, the training costs are those costs incurred by the training contractor in delivering the AAFT training and technical assistance (*e.g.*, trainers, logistics, travel expenses of trainees). A cost per therapist trained will be computed by taking the total cost of the training effort divided by the number of AAFT therapists trained.

### Analytic plan

#### Effectiveness-related analyses

Because of the multilevel nature of the data, Hierarchical Linear Modeling (HLM) [85], which is able to handle this type of data by allowing the relationship between the variables of interest to vary by higher-level groupings (*i.e.*, therapists and/or sites), will be used to analyze the effectiveness-related hypotheses. For H1.1 (*i.e.*, Target A-CRA is more likely for adolescents in the P4P group), the main independent variable is group assignment (IAU versus P4P), and the dependent variable is whether adolescents received Target A-CRA. Adolescents are at level one, therapists are at level two, and sites are at level three. For Hypothesis H1.2 (*i.e.*, Monthly A-CRA Competence is more likely for therapists in the P4P group), the main independent variable is group assignment (IAU versus P4P), and the dependent variable is the percentage of months therapists demonstrated A-CRA competence. Here, therapists are at the lowest level (*i.e.*, level one), and sites are at the next highest level (*i.e.*, level two). For Hypothesis H1.3 (*i.e.*, being in recovery after intake is more likely for adolescents in the P4P group), the main independent variable is group assignment (IAU versus P4P), and the dependent variable is whether adolescents are in recovery post-intake. Again, adolescents are at level one, therapists are at level two, and sites are at level three.

#### Cost effectiveness-related analyses

As is the usual case in CE analyses, we hypothesize that the experimental P4P group will be more expensive, but more effective relative to the IAU group. In order to test this general hypothesis, we relate the cost of reinforcement to its impact on each of the study's effectiveness-related hypotheses described in the previous section. In addition to noting whether the P4P group was statistically superior to the IAU group on each outcome, we will report the cost per adolescent receiving Target A-CRA, cost per month of demonstrated A-CRA competence, and cost per adolescent in recovery after intake. Using the notation of Glick and colleagues [86], the CE measure for the RTP study is the ratio of cost (*i.e.*, the difference between the average cost per individual in P4P and the average cost per individual in IAU, denoted by C) divided by effectiveness (*i.e.*, the comparable difference on effectiveness, denoted by Q). That is, CE = C/Q. Our basic CE measure is the CE of reinforcement using each of the measures in this study (*i.e.*, cost per adolescent who receives Target A-CRA; cost per month of demonstrated A-CRA competence; and cost per adolescent in recovery after intake). Each of these measures will be calculated as CE (P4P) = C (P4P)/Q(P4P). Here, C (reinforcement) is the difference in costs between the P4P and IAU groups, converted to the appropriate scale, and Q(P4P) is the corresponding difference in outcomes. Within CE measures, the numerator of each measure is the net cost (difference in cost per client and the grand mean), and the denominator is the net effectiveness (difference in the outcome per client and the grand mean) for each of the three respective outcomes (% of months of A-CRA/ACC competence; % of adolescents receiving Effective Threshold of A-CRA/ACC, and % of adolescents in recovery after intake).

## Discussion

The RTP study is one response to recommendations to examine the impact of P4P on improving the quality of care [87], and it represents the largest known randomized experiment to date to evaluate the impact of P4P methods at the staff level within the substance abuse treatment field. The study design was based on taking into consideration key P4P design elements as described by Rosenthal and Dudley [88], who have identified five key design elements of P4P programs. The following section briefly describes each of these elements and how they have been addressed in this study.

### 1. Individual versus group

The first element relates to whether the P4P initiative targets individuals or the organization. According to Rosenthal and Dudley [89], 14% of programs focused on individuals alone, 25% focused on both individuals and groups, and 61% focused on groups alone. However, consistent with their recommendation to provide incentives to the group or individual that is most responsible for the targeted behavior, therapists were selected as part of the RTP study given they are the ones who must ultimately implement the treatment with clients.

### 2. Paying the right amount

In order for an incentive to be effective, it must be commensurate with the costs in time and effort associated with achieving the targeted behavior. This is similar to the concept of financial salience being measured as part of the RTP study. Importantly, given the paucity of studies within the field of alcohol and drug treatment that have used P4P methods, determining appropriate incentive amounts was perhaps the most difficult aspect of designing the study. That is, incentive amounts selected had to simultaneously be large enough to significantly improve therapist performance, and small enough to be considered within a practical range for community-based treatment providers to implement.

Calculations suggested that full-time therapists in the P4P condition would earn on average an amount between $1,404 and $2,412 per 12-month period, which equated to approximately 4% to 7% of an average annual therapist salary of $35,000. While we believe these amounts are within a range that is practical for community-based treatment providers, the study will help us learn whether or not these incentives are large enough to impact performance.

### 3. Selecting high-impact performance measures

The third element relates to linking the incentives to performance measures that are meaningful and/or based upon sound scientific evidence and is similar to the concept of clinical relevance being measured as part of the RTP study. While research to date has provided only limited empirical support for the relationship between competency and outcomes, we believe this targeted behavior has considerable intuitive appeal and therefore will be perceived by therapists as being clinically relevant. Similarly, we believe therapists will find Target A-CRA to be a clinically meaningful performance measure, especially given the recent empirical evidence indicating that exposure to A-CRA procedures mediates the relationship between treatment retention and outcome [65].

### 4. Making payment reward all high-quality care

The fourth element relates to rewarding all who meet or exceed some threshold level of 'high quality care' as opposed to rewarding only the top performers (*e.g.*, top 10%)--the latter of which tends to create competition between providers and consequently decrease collaboration and sharing of ideas. Consistent with this recommendation, both Target A-CRA and Demonstration of Monthly A-CRA Competence represent threshold levels of high quality care, and achievement of one or both by one therapist does not reduce the opportunity for another therapist to also achieve the incentive.

### 5. Prioritizing quality improvement for underserved populations

The fifth element relates to reducing disparities in health and healthcare quality by offering relatively larger incentives for providing high-quality care to disadvantaged populations. Although the incentive amounts offered as part of the RTP did not differ for underserved populations, it may be possible to examine if there were differential rates of achievement of the targeted behaviors by race/ethnicity and/or gender.

### Study strengths and weaknesses

In addition to the use of random assignment, the RTP study has several other strengths. For example, a unique strength of the RTP study is the level of standardization in regard to the funding and training provided to the 29 participating agencies and their therapists. Specifically, because CSAT's approximately $30 million dollar AAFT initiative provided each of its grantees with close to $300,000 per year (for three years) as well as a comprehensive training and technical assistance package (via a separate training contract), the AAFT initiative provided an ideal opportunity to focus on examining the effectiveness and CE of P4P to improve EBT implementation and subsequent treatment outcomes for clients. Other strengths of the study include its: use of a theoretically-based conceptual framework; multi-site design; relatively large sample size; independent verification of therapist achievement of targeted behaviors; longitudinal assessment of therapist attitudes and client outcomes; inclusion of CE analyses; and hypothesis-driven multilevel analytic plan. Like all studies, however, the RTP study also has some limitations that must be acknowledged. First, although larger than any other known P4P experiment conducted to date, a greater number of sites and therapists would provide more statistical power and better generalizability. A second limitation of the study is that randomization was conducted by grantee rather than by therapist. However, as discussed previously, we believe the potential disadvantages associated with randomizing therapists within site (*e.g.*, compensatory rivalry, resentful demoralization) outweighed its advantages. Finally, because the targets being reinforced as part of this study are specific to the delivery of A-CRA procedures, the findings from this study may not generalize to other interventions and/or healthcare or behavioral health settings.

### Next steps

Although the recruitment and randomization of AAFT grantees has been completed, it is possible that additional therapists will be recruited as AAFT grantees hire new therapists. Indeed, this aspect of the RTP study is interesting in that in direct contrast to most studies, where attrition decreases statistical power, attrition actually has the potential to increase statistical power, given that therapists are typically replaced. Additionally, our research team continues to monitor therapist achievement of both Target A-CRA and Monthly A-CRA Competence and to administer both the therapist surveys and the TCAT. Given the study has just ended its first of three years, it will be some time before we are able to report on the impact of the incentives on therapist achievement of the targeted behaviors and on subsequent client outcomes. However, we plan to begin testing other parts of our conceptual framework. For example, we plan to examine the extent to which therapists' attitudes toward the incentives and TPB constructs explain variance in their intentions to achieve these behaviors. Given the increasing need to not only understand what interventions work, but how they work [89,90], research to understand the mechanisms through which reinforcing therapist performance via monetary incentives work is a critically important step.

## Competing interests

The authors declare that they have no competing interests.

## Authors' contributions

BRG conceived of and developed the study protocol, leads the study implementation, and drafted this manuscript. SHG, MDG, MLD, and DSS helped develop the study protocol and contributed to drafting this manuscript. All authors read and approved the final manuscript.
